# GRAM domain-containing protein 1B (GRAMD1B), a novel component of the JAK/STAT signaling pathway, functions in gastric carcinogenesis

**DOI:** 10.18632/oncotarget.23265

**Published:** 2017-12-15

**Authors:** Puja Khanna, Pei Jou Chua, Belinda Shu Ee Wong, Changhong Yin, Aye Aye Thike, Wei Keat Wan, Puay Hoon Tan, Gyeong Hun Baeg

**Affiliations:** ^1^ Department of Anatomy, Yong Loo Lin School of Medicine, National University of Singapore, Singapore 117594, Singapore; ^2^ Department of Pediatrics, New York Medical College, Valhalla, NY 10595, USA; ^3^ Division of Pathology, Singapore General Hospital, Singapore 169856, Singapore; ^4^ Academic Clinical Program for Pathology, Duke-NUS Graduate Medical School, Singapore 169857, Singapore

**Keywords:** JAK/STAT signaling, GRAMD1B, gastric cancer, apoptosis, immunohistochemistry

## Abstract

Dysregulated JAK/STAT signaling has been implicated in the molecular pathogenesis of gastric cancer. However, downstream effectors of STAT signaling that facilitate gastric carcinogenesis remain to be explored. We previously identified the *Drosophila* ortholog of human GRAMD1B in our genome-wide RNAi screen to identify novel components of the JAK/STAT signaling pathway in *Drosophila*. Here, we examined the involvement of GRAMD1B in JAK/STAT-associated gastric carcinogenesis. We found that GRAMD1B expression is positively regulated by JAK/STAT signaling and GRAMD1B inhibition decreases STAT3 levels, suggesting the existence of a positive feedback loop. Consistently, GRAMD1B and JAK/STAT signaling acted synergistically to promote gastric cancer cell survival by upregulating the expression of the anti-apoptotic molecule Bcl-xL. Interestingly, our immunohistochemical analysis for GRAMD1B revealed a gradual loss of cytoplasmic staining but an increase in the nuclear accumulation of GRAMD1B, as gastric tissue becomes malignant. GRAMD1B expression levels were also found to be significantly associated with clinicopathological features of the gastric cancer patients, particularly the tumor grades and lymph node status. Moreover, GRAMD1B and pSTAT3 (Tyr705) showed a positive correlation in gastric tissues, thereby confirming the existence of a close link between these two signaling molecules *in vivo*. This new knowledge about JAK/STAT-GRAMD1B regulation deepens our understanding of JAK/STAT signaling in gastric carcinogenesis and provides a foundation for the development of novel biomarkers in gastric cancer.

## INTRODUCTION

Gastric cancer continues to be one of the most predominant cancers worldwide [1, 2]. Several risk factors such as *Helicobacter pylori* infection, dietary habits, lifestyles and demographics contribute to the development and spread of the malignancy [3]. Over the years, decreased incidence rates, better treatment strategies and increased awareness have contributed to the reduction of gastric cancer incidence [4], but the mortality rates continue to be alarming. Studies on the genomic landscape of gastric carcinoma have led to the identification of several molecular targets and signaling molecules involved in the process of gastric tumorigenesis [5, 6]. Particularly, epidermal growth factor receptor family (ErbB) members [7–11], vascular endothelial growth factor receptor family (VEGFR) members [12–14] and PI3K/Akt/mTOR pathway components [15, 16] were found to be involved in the molecular pathogenesis of gastric cancer. However, drugs targeting these signaling molecules have failed to show promise in clinical trials, and thus there continues to be a need to identify alternative molecules that can be targeted clinically [17–20].

The JAK/STAT (Janus Kinase/Signal Transducer and Activator of Transcription) cascade is the principal signal transduction pathway in cytokine and growth factor signaling [21–23]. Tightly regulated JAK/STAT signaling is of utmost importance in regulating cellular processes such as cellular proliferation, differentiation, migration and survival [23, 24], as dysregulation of the signaling is closely associated with various human diseases. In particular, numerous studies have shown that JAK/STAT signaling contributes to the process of tumorigenesis in a wide variety of haematological malignancies and solid tumors [25]. Constitutively-active STAT3 has been found in several gastric cancer cell lines, and its inhibition by the ectopic expression of dominant negative STAT3 or JAK inhibitors resulted in apoptosis of these cancer cells, suggesting that altered JAK/STAT signaling plays an important role in gastric carcinogenesis [26–28]. In support of this, immunohistochemical analysis of gastric adenocarcinoma tissues showed that STAT3 expression is closely associated with tumour, node and metastasis (TNM) stage as well as survival, suggesting that it functions as a biomarker predicting poor prognosis of gastric cancer [29]. Hence, targeting the components of the JAK/STAT signaling pathway holds great potential in the treatment of gastric cancer [30]. However, the mechanisms underlying gastric cancer utilized by JAK/STAT signaling is still not fully understood. In particular, the downstream effectors of JAK/STAT signaling that transduce the extracellular cues to promote gastric carcinogenesis remain to be elucidated.

In our previous study for the identification of additional JAK/STAT signaling pathway components in *Drosophila* [31], we identified the *Drosophila* ortholog of GRAMD1B (GRAM domain-containing protein 1B), an uncharacterized protein belonging to the GRAM domain family of proteins [32]. The GRAM domain is an intracellular protein-binding or lipid-binding signaling domain [33]. In myotubularin, mutations in the GRAM domain were shown to disrupt its phosphatase activity and lead to X-linked myotubular myopathy, suggesting the importance of GRAM domain [32]. Furthermore, another member of the GRAM domain family, GRAMD4 was shown to act in p73-mediated apoptosis [34]. However, functions for most of these family members including GRAMD1B are still unknown. More recently, a few reports implicated GRAMD1B in tumorigenesis. Specifically, GRAMD1B was reported to be involved in chemoresistance of ovarian cancer patients, and silencing of this gene led to a synergistic anti-tumor effect in combination with paclitaxel [35].

In this study, we examined the functional relevance of JAK/STAT-GRAMD1B interaction in gastric cancer. GRAMD1B expression was positively regulated by JAK/STAT signaling which in turn functioned to regulate STAT3 levels, suggesting the existence of a positive feedback loop. Our study also showed that GRAMD1B, together with JAK/STAT signaling, facilitates gastric cancer cell survival by modulating the expression of anti-apoptotic genes such as *Bcl-xL*. Interestingly, our immunohistochemical analysis for GRAMD1B using 63 paired gastric cancer tissue microarrays (TMAs) revealed decreased cytoplasmic but increased nuclear staining of GRAMD1B as tissue becomes malignant, implying the importance of nuclear GRAMD1B localization in gastric tumorigenesis. Importantly, a case-wise comparison between the expression of GRAMD1B and pSTAT3 (Tyr705) in gastric tissue showed a positive correlation. Our study suggests that GRAMD1B may play an essential role in JAK/STAT-associated gastric carcinogenesis.

## RESULTS

### JAK/STAT signaling regulates *CG34394* transcription in the *Drosophila* S2 cell line

**Figure 1:**
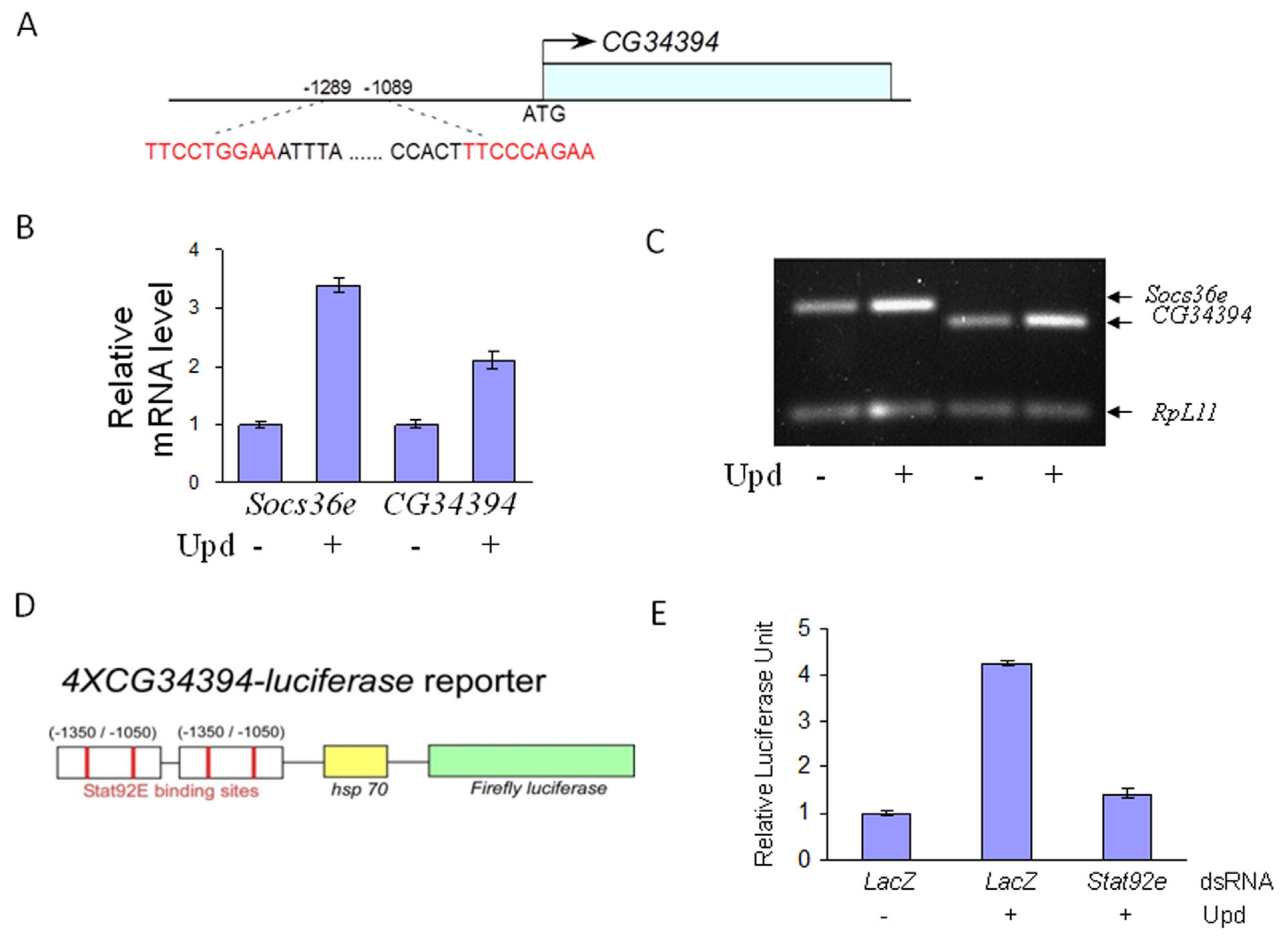
JAK/STAT signaling regulates *CG34394* transcription in the *Drosophila* S2 cells **(A)** *CG34394* genomic region contains two potential STAT92E binding sites. **(B** and **C)** qRT-PCR analysis shows that transcription of *CG34394* and *Socs36e* (a positive control) is upregulated by Upd stimulation. **(D)** Two tandem repeats of the *CG34394* genomic fragment were placed upstream of cDNA encoding the firefly *luciferase* gene to construct a *4XCG34394-luciferase* reporter. **(E)** The reporter activity is induced by Upd stimulation, but knockdown of *Stat92e* negated the reporter activity.

We have previously identified CG34394 as a novel component of the JAK/STAT signaling pathway in *Drosophila* [31]. Interestingly, *in-silico* analysis of the genomic region of *CG34394* revealed two potential STAT92E (the sole STAT in *Drosophila*) binding sites (Figure 1A), suggesting that STAT92E regulates the transcription of *CG34394*. To test this hypothesis, we carried out cellular assays using the cytokine-like molecule Unpaired (Upd) as a JAK/STAT signaling inducer in the *Drosophila* S2 cell line. Real time qRT-PCR analysis showed an increase in *CG34394* mRNA levels upon Upd stimulation, suggesting that its transcription is positively regulated by STAT92E (Figure 1B-1C). *Socs36e* (the *Drosophila* homolog of *SOCS*) mRNA levels serve as a positive control. Since the promoter region of *CG34394* contains two STAT92E binding sites, we generated a reporter by placing two tandem repeats of *CG34394* genomic fragment (−1350/−1050) upstream of a minimal heat-shock promoter-driven cDNA encoding firefly *luciferase* gene, referred to as *4XCG34394-luciferase* (Figure 1D). If the reporter is responsive to JAK/STAT signaling, the reporter activity will be increased by Upd, but the Upd-induced reporter activity will be decreased by STAT92E inhibition. We found an almost 4-fold induction of the reporter activity upon Upd stimulation, whereas the addition of dsRNA-*Stat92e* blocked the reporter activity back to the level observed in cells without Upd (Figure 1E). This suggests that *CG34394* is a STAT92E downstream target in *Drosophila*.

### JAK/STAT signaling regulates GRAMD1B expression in gastric cancer cell lines

**Figure 2:**
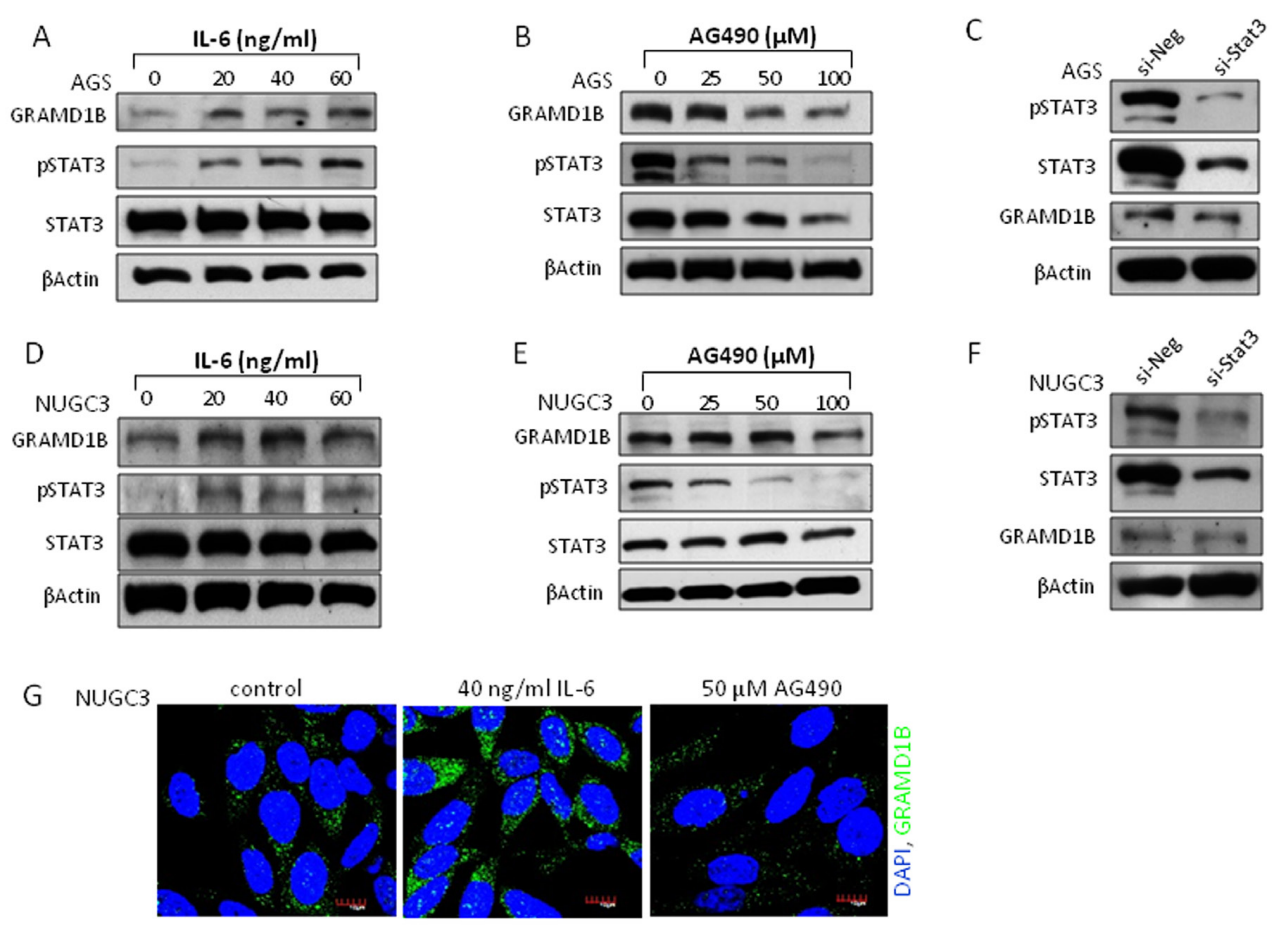
JAK/STAT signaling regulates GRAMD1B expression in gastric cancer cell lines The gastric cancer cell lines AGS **(A-C)** and NUGC3 **(D-G)** were used. (A, B, D and E) IL-6-induced JAK/STAT signaling increases GRAMD1B levels but JAK/STAT signaling inhibition by the JAK2 inhibitor AG490 decreases GRAMD1B levels in a dose-dependent manner. (C and F) Decreased GRAMD1B expression is also observed upon *Stat3* knockdown. (G) Immunofluorescence assay shows the regulation of GRAMD1B expression by JAK/STAT signaling. (Scale bar = 10μm).

*Drosophila* CG34394 encodes a protein, which contains a GRAM domain and shows high level of conservation with human GRAMD1B protein, confirming a high level of similarity between CG34394 and GRAMD1B. To investigate if JAK/STAT-mediated transcriptional regulation of *CG34394* is conserved across phyla, we examined the expression of GRAMD1B using the human gastric cancer cell lines AGS and NUGC3. A dose-dependent increase in GRAMD1B of 86 kDa, which is considered isoform 1 (NCBI: NP_001273492.1), was observed upon the treatment of IL-6, an inducer of JAK/STAT signaling in AGS cells (Figure 2A). We next examined the inhibitory effect of JAK/STAT signaling on GRAMD1B expression by treating cells with the JAK2 inhibitor AG490 at various concentrations, and found that GRAMD1B levels decrease by AG490 in a dose-dependent manner (Figure 2B). To further confirm these findings, we treated AGS cells with siRNA for *Stat3*, which is a member of the STAT family and plays a key role in many cellular processes such as cell growth and apoptosis. As expected, we observed a decrease in GRAMD1B levels on *Stat3* knockdown (Figure 2C). Its expression was also increased in response to IL-6, and decreased upon AG490 or si-Stat3 treatment in NUGC3 cells (Figure 2D-2G), suggesting that the regulation of GRAMD1B expression by JAK/STAT signaling is not cell type-dependent. Taken together, our data suggests that GRAMD1B is a *bona-fide* downstream target of JAK/STAT signaling across phyla.

### GRAMD1B inhibition decreases STAT3 levels

**Figure 3:**
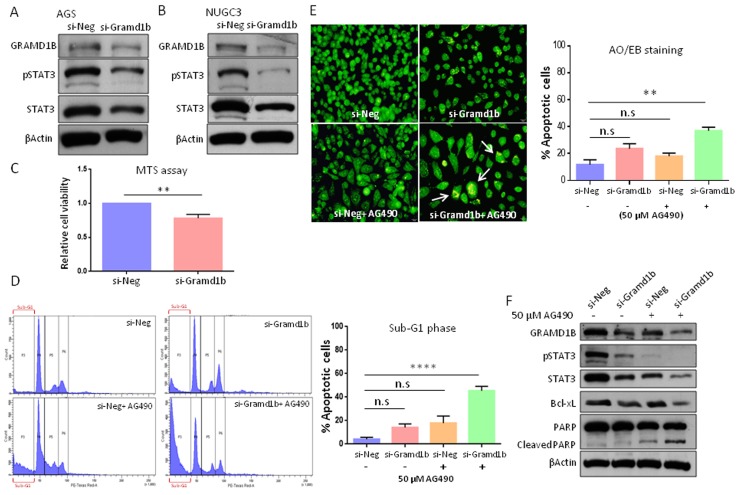
GRAMD1B and JAK/STAT signaling act synergistically to regulate anti-apoptotic gene expression **(A** and **B)** Knockdown of *Gramd1b* causes a decrease in both total STAT3 and pSTAT3 (Tyr705) levels in AGS and NUGC3 cells. **(C-F)** Cellular assays were conducted using AGS cells. (C) MTS assay reveals that *Gramd1b* knockdown decreases cell growth by approximately 20% compared to control. (D) PI staining suggests the synergistic effects of GRAMD1B and JAK/STAT signaling on cell survival. (E) AO/EB staining suggests that inhibition of both GRAMD1B and JAK/STAT signaling synergistically increases the number of apoptotic cells (white arrows). (F) Decreased Bcl-xL expression on *Gramd1b* knockdown is further enhanced by co-treatment with AG490. The increase in cleaved PARP levels on *Gramd1b* knockdown is also further enhanced by co-treatment with AG490. ^**^, *P<0.01*; ^****^, *P<0.0001.*

Many interferon-stimulated genes (ISGs) encode products that feedback into the JAK/STAT circuitry to positively or negatively affect the signaling activity [36, 37]. The SOCS family of proteins are well-known to be induced by cytokines, but upon induction they feedback into the JAK/STAT pathway to inhibit signaling by either blocking STAT recruitment to the cognate receptor or by promoting ubiquitination and degradation of the JAK/receptor complex [38]. To test whether GRAMD1B functions in a similar feedback manner, we examined the inhibitory effect of GRAMD1B on JAK/STAT signaling. Knockdown efficiency of si-Gramd1b was optimized by Western blot analysis (data not shown). Interestingly, we observed decreased levels of total STAT3 and pSTAT3 (Tyr705) levels in AGS cells transfected with si-Gramd1b (Figure 3A). To confirm these results, we also knock-downed *Gramd1b* in NUGC3 cells and observed a similar decrease in STAT3 levels (Figure 3B). To rule out off-target effects, we also inhibited GRAMD1B in AGS cells using a second siRNA (si-Gramd1b-2) and confirmed a decrease in total and phosphorylated STAT3 (Supplementary Figure 1). These findings suggest that GRAMD1B acts as a positive regulator of JAK/STAT signaling in gastric cancer cell lines.

### GRAMD1B functions in JAK/STAT-associated anti-apoptotic gene expression

Dysregulated JAK/STAT signaling resulting from constitutively-active STAT3 or its downstream targets has been implicated in gastric tumorigenesis [27]. Since GRAMD1B is a novel downstream target of the JAK/STAT cascade, we first explored its role in the cell growth of gastric cancer cells. MTS cell proliferation assay revealed that cell growth of AGS cells decreases about 20% by *Gramd1b* knockdown, compared to control (Figure 3C). Several studies have suggested the crucial role of STAT3 in gastric cancer cell survival, such that loss of STAT3 led to cell cycle arrest in the G1 phase [39, 40]. Interestingly, propidium iodide (PI) staining revealed an increase in the number of apoptotic cells in the sub-G1 phase on siRNA-mediated knockdown of *Gramd1b*, and this increase was further enhanced on AG490 co-treatment (Figure 3D), suggesting the synergistic role of GRAMD1B and JAK/STAT signaling in apoptosis. We further validated this synergistic effect using the Acridine orange (AO)/ Ethidium bromide (EB) staining assay. Higher percentage of cells was found to be positive for apoptosis on co-treatment with si-Gramd1b and AG490, compared to treatment with si-Gramd1b or AG490 alone (Figure 3E). To examine the possible mechanism underlying the increase in apoptosis on GRAMD1B inhibition, we next examined the inhibitory effects of GRAMD1B on apoptosis-related gene expression as JAK/STAT signaling is known to promote cell growth by promoting the expression of anti-apoptotic genes such as *Bcl-xL*, *Mcl-1* and *Survivin* [41]. We found that treatment of cells with AG490 caused a decrease in the expression of Bcl-xL by approximately 15% compared to control. However, co-treatment of the cells with AG490 and si-Gramd1b decreased the expression of Bcl-xL by almost 75%. Similarly, a slight increase in cleaved PARP levels in AG490 treated cells was observed, but upon co-treatment with si-Gramd1b this was further enhanced reflecting an increase in apoptosis (Figure 3F).

### High levels of nuclear GRAMD1B is associated with aggressive diffuse-type of gastric cancer

**Figure 4:**
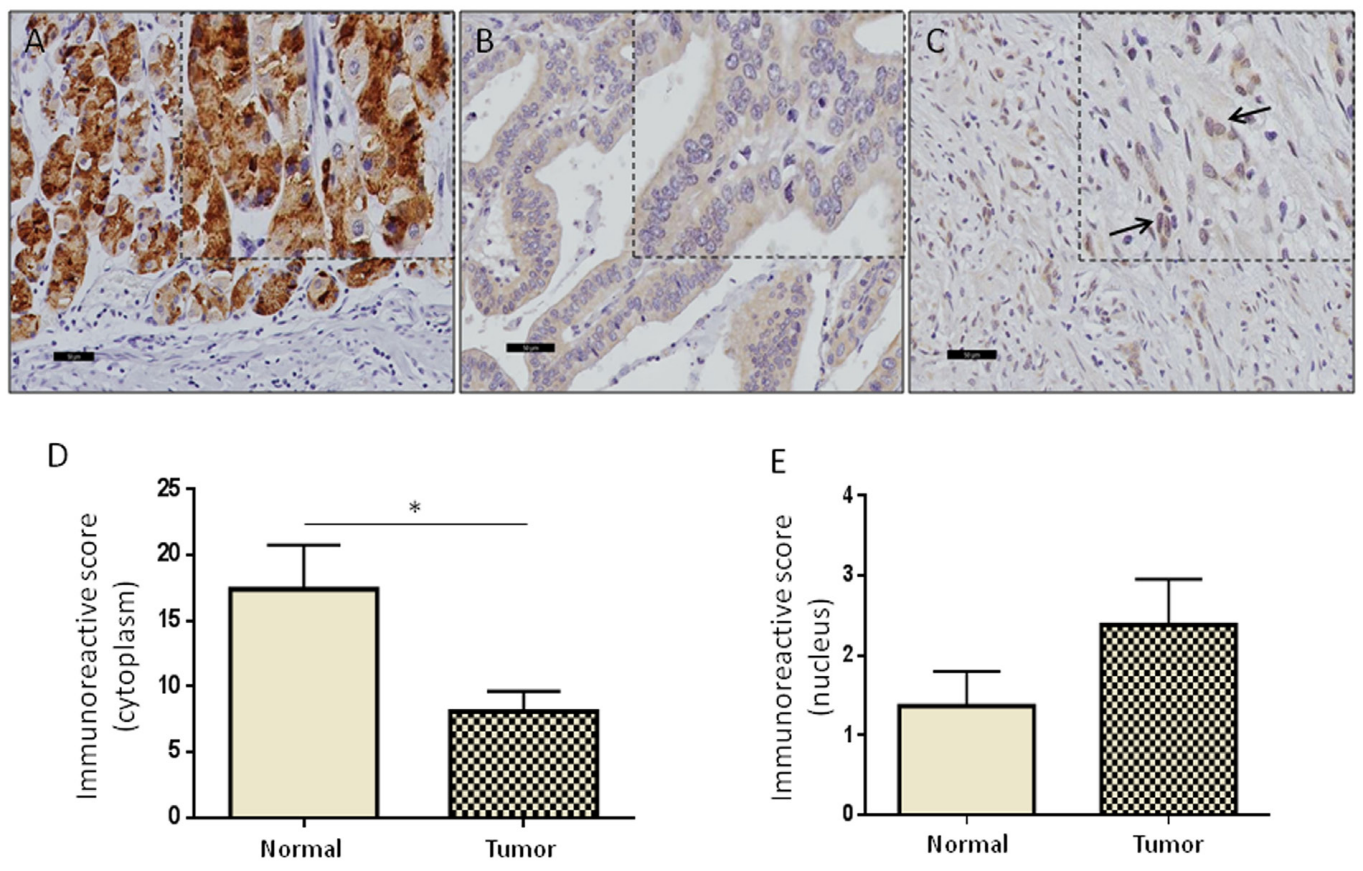
High levels of nuclear GRAMD1B are associated with aggressive diffuse-type of gastric cancer **(A)** High levels of cytoplasmic GRAMD1B staining are observed in normal gastric tissue. **(B** and **C)** Decreased cytoplasmic staining is observed in the intestinal-type of gastric cancer, and elevated nuclear staining of GRAMD1B is detected in the aggressive diffuse-type of gastric cancer. (A-C) Scale bar = 50μm. **(D** and **E)** A case-wise comparison of the immunoreactive scores for GRAMD1B in normal gastric tissue versus gastric tumor tissue shows a significant decrease in cytoplasmic staining (*P=0.02*) and an increase in nuclear staining (*P=0.176*). ^*^, *P<0.05.*

To further investigate the role of GRAMD1B in oncogenesis, we performed immunohistochemical analyses for GRAMD1B in 63 human gastric cancer tissue with matched normal tissue samples. The clinicopathological parameters of the patient cohort used for the study are listed in Supplementary Table 1. GRAMD1B was found to be localized in both cytoplasm and nucleus of normal and tumor tissues. However, we noticed that there was a gradual decrease in cytoplasmic GRAMD1B but an increase in nuclear GRAMD1B as normal gastric tissue turns into aggressive diffuse-type of gastric cancer (Figure 4A-4C). A case-wise comparison of cytoplasmic and nuclear staining of tumor tissues versus matched normal tissues confirmed the decrease in cytoplasmic staining (*P*=0.02) (Figure 4D) and the increase in nuclear staining (*P*=0.176) of GRAMD1B (Figure 4E). The expression level of GRAMD1B was represented as the immunoreactive score (IRS), which takes into account both the percentage of stained cells as well as the intensity of staining. The mean IRS was set as the cut-off to classify GRAMD1B immunostaining into high and low groups. To determine the correlation between GRAMD1B expression levels and clinicopathological parameters of gastric cancer patients, univariate statistical analysis was carried out and outcome is summarized in Table 1. In brief, lower cytoplasmic GRAMD1B staining was associated with higher tumor grades (*P*=0.026) and lymph node involvement (*P*=0.005). Particularly, intestinal-type of gastric cancer showed higher cytoplasmic staining as compared to the aggressive diffuse-type of gastric cancer (*P*=0.009). Concurrently, the diffuse-type of gastric cancer showed significantly higher amounts of nuclear staining as compared to the intestinal-type of gastric tumor (*P*=0.033), suggesting that nuclear localization of GRAMD1B may play an important role in the diffuse-type of gastric cancer.

**Table 1:** Clinicopathological significance of GRAMD1B in gastric cancer

Clinicopathological parameters	IRS_cytoplasm <= mean	IRS_cytoplasm > mean	P value	IRS_nucleus <= mean	IRS_nucleus > mean	P value
	**n (%)**	**n (%)**		**n (%)**	**n (%)**	
**Gender**						
**Male**	24 (57.1%)	18 (42.9%)	0.027^*^	31 (73.8%)	11 (26.2%)	1
**Female**	18 (85.7%)	3 (14.3%)		15 (71.4%)	6 (28.6%)	
**Age (Years)**						
**<=65**	23 (74.2%)	8 (25.8%)	0.287	22 (71.0%)	9 (29.0%)	0.782
**66+**	19 (59.4%)	13 (40.6%)		24 (75.0%)	8 (25.0%)	
**Grade**						
**G <=2**	11 (47.8%)	12 (52.2%)	0.026^*^	17 (73.9%)	6 (26.1%)	1
**G >2**	31 (77.5%)	9 (22.5%)		29 (72.5%)	11 (27.5%)	
**Lymph Node status**						
**pN0**	8 (38.1%)	13 (61.9%)	0.005^**^	15 (71.4%)	6 (28.6%)	0.324
**pN1**	10 (76.9%)	3 (23.1%)		9 (69.2%)	4 (30.8%)	
**pN2**	12 (75%)	4 (25.0%)		10 (62.5%)	6 (37.5%)	
**pN3**	12 (92.3%)	1 (7.7%)		12 (92.3%)	1 (7.7%)	
**Extent**						
**pT<=1**	5 (45.5%)	6 (54.5%)	0.157	7 (63.6%)	4 (36.4%)	0.469
**pT>1**	37 (71.2%)	15 (28.8%)		39 (75.0%)	13 (25.0%)	
**WHO classification**						
**Signet Ring**	13 (86.7%)	2 (13.3%)	0.331	8 (53.3%)	7 (46.7%)	0.237
**Adenocarcinoma**	10 (55.6%)	8 (44.4%)		15 (83.3%)	3 (16.7%)	
**Tubular**	12 (60.0%)	8 (40.0%)		14 (70.0%)	6 (30.0%)	
**Mucinous**	1 (100.0%)	0 (0%)		1 (100%)	0 (0.0%)	
**Mixed**	6 (66.7%)	3 (33.3%)		8 (88.9%)	1 (11.1%)	
**Lauren classification**						
**Diffuse**	17 (81.0%)	4 (19.0%)	0.009^**^	11 (52.4%)	10 (47.6%)	0.033^*^
**Intestinal**	15 (48.4%)	16 (51.6%)		26 (83.9%)	5 (16.1%)	
**Mixed**	10 (90.9%)	1 (9.1%)		9 (81.8%)	2 (18.2%)	
**Ming classification**						
**Infiltrative**	39 (69.6%)	17 (30.4%)	0.209	40 (71.4%)	16 (28.6%)	0.663
**Expansive**	3 (42.9%)	4 (57.1%)		6 (85.7%)	1 (14.3%)	
**Stromal reaction**						
**No**	11 (61.1%)	7 (38.9%)	0.568	13 (72.2%)	5 (27.8%)	1
**Yes**	31 (68.9%)	14 (31.1%)		33 (73.3%)	12 (26.7%)	
**LVI**						
**Absent**	18 (66.7%)	9 (33.3%)	1	19 (70.4%)	8 (29.6%)	0.777
**Present**	24 (66.7%)	12 (33.3%)		27 (75.0%)	9 (25.0%)	
**PNI**						
**Absent**	20 (62.5%)	12 (37.5%)	0.595	23 (71.9%)	9 (28.1%)	1
**Present**	22 (71.0%)	9 (29.0%)		23 (74.2%)	8 (25.8%)	
**Perforation**						
**No**	41 (67.2%)	20 (32.8%)	1	44 (72.1%)	17 (27.9%)	1
**Yes**	1 (50.0%)	1 (50.0%)		2 (100.0%)	0 (0.0%)	

### Expression of GRAMD1B and pSTAT3 (Tyr705) shows a positive correlation

**Figure 5:**
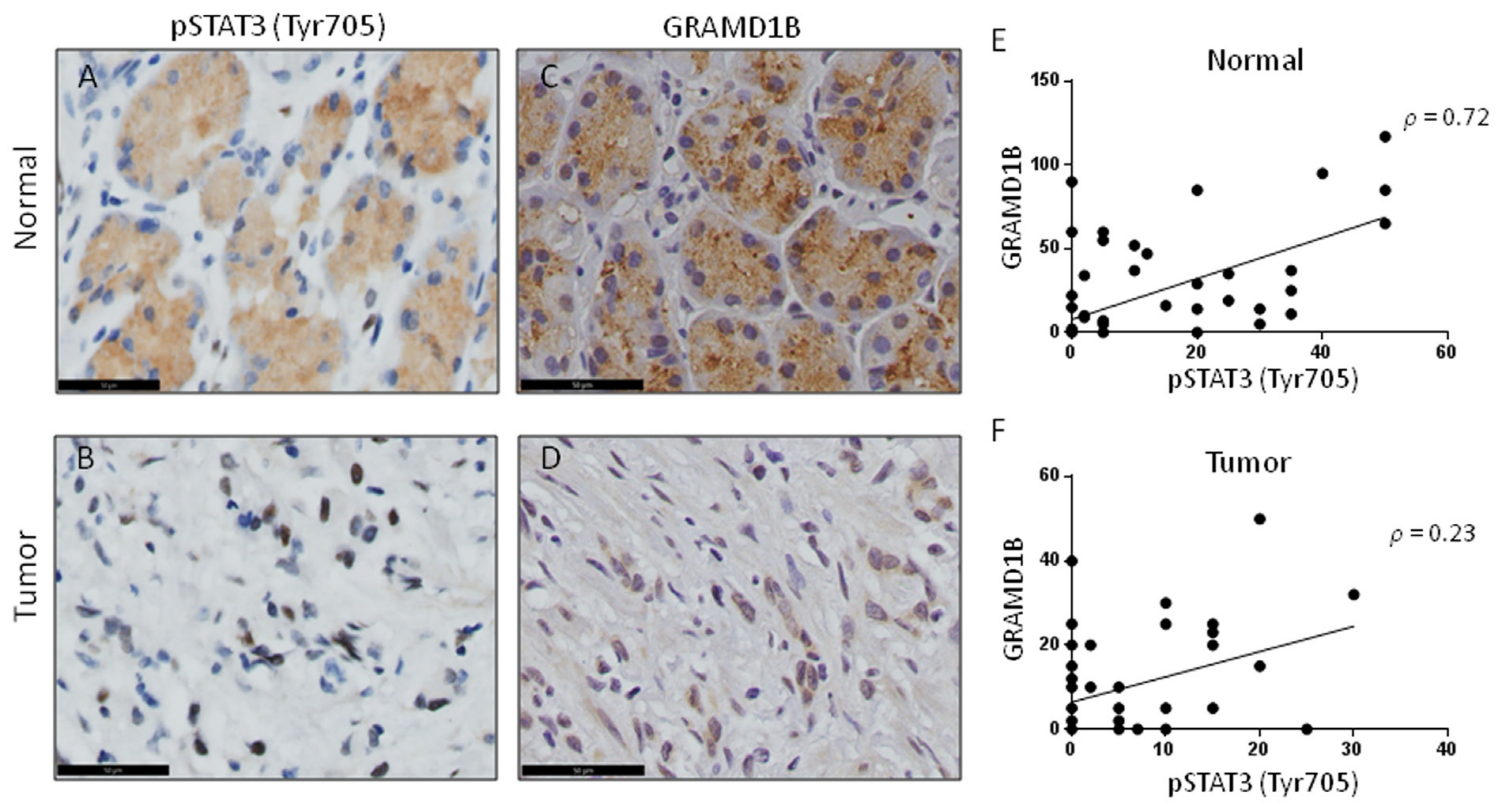
GRAMD1B expression pattern shows a positive correlation with pSTAT3 (Tyr705) in gastric tissue **(A)** Normal gastric tissue shows high levels of cytoplasmic pSTAT3, **(B)** whereas gastric tumor tissue shows a nuclear accumulation of pSTAT3. **(C)** Immunohistochemical analyses for GRAMD1B also reveal cytoplasmic staining in normal gastric tissue, **(D)** but show nuclear staining in gastric tumor tissue. (A-D) Scale bar = 50μm. **(E)** A positive correlation is observed between GRAMD1B and pSTAT3 staining in normal gastric tissue (Spearman’s correlation coefficient: 0.72, *P<0.0001*), **(F)** as well as in gastric tumor tissue (Spearman’s correlation coefficient: 0.23, *P=0.08*).

Upon activation, cytoplasmic STAT3 dimerizes and translocates into the nucleus to facilitate the transcription of their downstream target genes, suggesting the important role of nuclear STAT3 in tumorigenesis [24]. Our immunohistochemical staining of matched normal and tumor gastric tissue samples indeed revealed a decrease in cytoplasmic pSTAT3 but an increase in nuclear pSTAT3 levels in gastric tumor tissue samples (Figure 5A-5B). This finding is in accordance with previous reports [26, 42], and is similar to our observation that nuclear GRAMD1B accumulates in tumor tissues (Figure 5C-5D). In support of this, on a case-wise comparison of GRAMD1B and pSTAT3 expression for the tissue samples, we found the existence of a positive correlation for staining in normal gastric tissues with a Spearman’s correlation coefficient of 0.72 (*P*<0.0001) (Figure 5E). Similarly, a positive correlation for GRAMD1B and pSTAT3 staining was observed in tumor gastric tissues with a Spearman’s correlation coefficient of 0.23 (*P*=0.08) (Figure 5F). These findings further support the existence of a close association between GRAMD1B and STAT3 *in vivo*.

## DISCUSSION

The JAK/STAT cascade is a fundamental signal transduction pathway that is primarily responsible for cytokine and growth factor signaling, and functions in a wide range of cellular processes such as immune response and cell growth [22, 24, 43]. Hence, dysregulation of this pathway has been associated with a wide variety of human diseases such as immune disorders and cancer. For instance, JAK/STAT signaling has been implicated in gastric tumorigenesis. Persistently-active STAT3 was found in several gastric cancer cell lines, where it serves a key mediator of cancer growth and metastatic potential [26, 39, 44]. Analyses with human gastric tumor tissue have also shown the association of STAT3 with many clinicopathological features, including TNM staging and survival, thereby establishing it as an important prognostic marker in gastric tumors [29, 45]. However, the exact molecular mechanisms of how it promotes tumorigenesis still remain to be elucidated.

In this study, we demonstrated that GRAMD1B expression is regulated by JAK/STAT signaling both in *Drosophila* and humans, and conversely GRAMD1B positively regulates STAT3 levels, suggesting that a positive-feedback loop occurs. Our study also showed potential oncogenic role of GRAMD1B in gastric tumor, together with JAK/STAT signaling by enhancing anti-apoptotic gene expression. Interestingly, our immunohistochemical analyses of human gastric tumor tissues uncovered a decreased cytoplasmic but an increased nuclear GRAMD1B staining in the aggressive diffuse-type of gastric cancer. Moreover, GRAMD1B expression showed a strong positive correlation with pSTAT3 expression in gastric tissues. These findings suggest that GRAMD1B plays important roles in JAK/STAT-associated gastric cancer and that it may serve as a novel diagnostic biomarker in gastric cancer.

### GRAMD1B expression is regulated by JAK/STAT signaling

STAT dimers in the nucleus bind to specific regulatory sequences to activate or repress transcription of their target genes [46]. *In silico* analysis of the promoter region of *Drosophila CG34394* revealed the presence of potential STAT92E binding sites (TTCNNNGAA). In support of this, we detected increased mRNA levels of *CG34394* upon Upd stimulation. Furthermore, Upd induction enhanced the *4XCG34394-luciferase* reporter activity in a STAT92E-dependent manner, suggesting that *CG34394* is a *bona-fide* STAT92E downstream target in *Drosophila*. To examine if this transcriptional regulation is evolutionarily conserved in mammals, we tested the expression of GRAMD1B in gastric cancer cell lines, and found that its levels are induced by IL-6 stimulation but suppressed by the JAK2 inhibitor AG490. Interestingly, a study showed that GRAMD1B levels increase on treatment with IFN- β, an inducer of JAK/STAT signaling [47]. Taken together, our results suggest that GRAMD1B is a novel downstream target of JAK/STAT signaling across phyla.

### GRAMD1B regulates JAK/STAT signaling

Notably, GRAMD1B inhibition resulted in a reduction of total STAT3 as well as pSTAT3 levels, suggesting that a feedback loop between GRAMD1B and JAK/STAT signaling occurs. It is well known that a number of the STAT targets on activation, feedback into the JAK/STAT circuitry and affect the signaling activity [22, 48]. The best studied are the SOCS family of proteins, a class of cytokine-inducible inhibitors of JAK/STAT signaling. Cytokine stimulation increases expression of these SH2 domain-containing signaling molecules, which feedback into the pathway to interrupt the signaling [37, 49]. Another IFN-stimulated ubiquitin-like protein, ISG15 was found to be associated with enhanced and prolonged JAK/STAT signaling, suggesting that ISG15 in return positively affects JAK/STAT signaling [50].

It is widely accepted that soluble STAT3 molecules on activation transit freely into the nucleus to activate the transcription of target genes, however, there is now increasing evidence that suggests the existence of a membrane-associated transportation system for STAT3 [48, 51]. The “signaling endosome hypothesis” supports the occurrence of an active, directed signal transduction process *via* the cytoskeletal transport apparatus [52]. In particular, active cytoplasmic transport of STAT3 was found to be dependent on growth factor-induced receptor-mediated endocytosis, such that STAT3 co-localized with receptor-ligand complexes in these endocytic vesicles, thereby transiting from the plasma membrane to the nucleus. Furthermore, the disruption of endocytosis was found to prevent STAT3 nuclear translocation and abrogate STAT3-mediated gene transcription [53], providing evidence for the importance of endocytosis in STAT3 signaling. IL-6-mediated association of STAT3 with clathrin heavy chain and other protein binding partners in early endosomes, further emphasizes a vital contribution of the endocytic pathway in productive IL-6/STAT3 signaling [51]. Our results revealed a decrease in STAT3 and pSTAT3 levels on GRAMD1B inhibition. As the GRAM domain has been predicted to be involved in protein/lipid-binding membrane-associated processes [33], it is conceivable that GRAMD1B may serve as an interacting partner in the endocytic pathway, thereby required for the stabilization and/or trafficking of STAT3. Another member of the GRAM domain containing family of proteins, myotubularin has also been implicated in the functioning of late endosomal trafficking and vacuolar morphology *via* its interaction with phosphatidylinositol 3,5-bisphosphate [54], suggesting the potential role of the GRAM domain in membrane-associated signal transduction.

Immunohistochemical studies with gastric tumor tissues have revealed pSTAT3 expression in the nucleus, with higher expression levels in advanced gastric tumors [26, 42]. Interestingly, we also detected nuclear GRAMD1B expression in our tumor samples, with the more aggressive diffuse-type of gastric cancer showing higher expression. Similarity in the expression patterns and levels for these signaling molecules further suggests a close link between these signaling molecules *in vivo*.

### GRAMD1B promotes cell survival

Several gastric cancer cell lines showed the presence of constitutively-active STAT3, which functions to facilitate cell survival *via* upregulating the expression of its downstream target genes such as *Survivin* and *Cyclin D1* [26]. More recently, the JAK/STAT signaling cascade was also found to regulate gastric cancer growth and survival *via* cell apoptosis and cell cycle shift induction, such that STAT3 inhibition increases apoptosis and arrests cells in the G1 phase [40]. Another study implicated the role of STAT3 signaling in angiogenesis of gastric tumors by regulation of its target genes *cyclin D1*, *Bcl-xL* and *VEGF* [39]. Since GRAMD1B is a JAK/STAT downstream target, we explored the role of GRAMD1B in the process of JAK/STAT-associated cell survival. PI and AO/EB staining revealed an increase in the number of apoptotic cells on siRNA-mediated knockdown of *Gramd1b*, and this increase was further enhanced on AG490 co-treatment. These synergistic effects were also detected in the regulation of anti-apoptotic gene expression. Another member of the GRAM domain containing family of proteins, GRAMD4 has also been found to promote p73-induced apoptosis by interacting with Bcl-2 and promoting Bax mitochondrial relocalization [34]. Taken together, our study may suggest the important role of GRAMD1B in gastric cancer survival, together with JAK/STAT signaling *via* modulating anti-apoptotic gene expression.

### GRAMD1B promotes gastric tumorigenesis

Immunohistochemical analyses of matched normal and tumor gastric tissues revealed a decrease in GRAMD1B cytoplasmic staining but an increase in its nuclear staining as normal gastric tissue becomes aggressive diffuse-type of gastric cancer. GRAMD1B expression levels were also found to be reflective of several clinicopathological parameters, including tumor grade and lymph node status. Specifically, decreased cytoplasmic expression of GRAMD1B was associated with higher tumor grades and lymph node involvement. Consistently, decreased cytoplasmic but increased nuclear GRAMD1B levels were detected in the more aggressive diffuse-type of gastric cancer as compared to the intestinal-type, suggesting the potential role of nuclear GRAMD1B in gastric tumor progression. It is worth to note that translocation of pSTAT3 to the nucleus is also highly associated with several tumor parameters, including TNM stage and survival. Hence, the occurrence of a similar translocation-based phenomenon for GRAMD1B and pSTAT3 may suggest the possibility of them acting concurrently to promote gastric tumorigenesis. STAT3 is also involved in several other hallmarks of gastric cancer that include cell migration and invasion [27–29]. Hence exploring the function of GRAMD1B in these processes also holds promise in deciphering the exact role of GRAMD1B in gastric tumorigenesis.

## MATERIALS AND METHODS

### Tissue specimens and cell culture

Paraffin-embedded TMAs of tumor tissues with adjacent normal tissues were obtained from 63 gastric cancer patients from Singapore General Hospital, Singapore. Institutional Review Board approval (CIRB 2007/104/F) was obtained for the study. The human gastric cancer cell lines AGS and NUGC3 were obtained from American Type Culture Collection (ATCC, Rockville, MD), and maintained in RPMI-1640 containing 2.05mM L-glutamine (HyClone^TM^) supplemented with 10% FBS (Gibco) and 1% Penicillin-Streptomycin (Gibco). The *Drosophila* S2 cell line was maintained in Schneider medium (Gibco) with 10% FBS and 1% Penicillin-Streptomycin in a 25°C incubator.

### Chemicals, vectors and transfections

IL-6 (PeproTech, USA) was reconstituted in water, and cells were stimulated for 6 hours in RPMI-1640. The JAK2 inhibitor AG490 (Sigma-Aldrich) was reconstituted in DMSO, and cells were treated with AG490 for 24 hours prior to harvest. si-Gramd1b (Dharmacon, custom siRNA Sense: 5′CCAAAGAGACAUUCUCCUU dTdT 3′ Antisense: 5′ AAGGAGAAUGUCUCUUUGG dTdT 3′), si-Gramd1b-2 (Ambion, #AM16708) and ON-TARGETplus *Stat3* (Dharmacon, #L-003544-00) were used to carry out knockdown studies *in vitro*. Non-targeting siRNA (Ambion, #4390843) was used as a control. Cells were transfected using Lipofectamine 3000 reagent (Invitrogen, USA) in antibiotic-free RPMI-1640 medium containing 10% FBS.

### Reporter construction and luciferase assay

The promoter region of *CG34394* containing potential STAT92E-binding sites was amplified by PCR, using two different sets of oligos: (1) ATA CTG CAG ATT GAA ATT CAC AAC GAA ATT CAG TGT TCA (PstI), AAT GAA TTC CAT TCG CCA TTA CAT ACC ATT TTA ATT GAC (EcoRI); (2) ATA AGATCT ATT GAA ATT CAC AAC GAA ATT CAG TGT TCA (BglII), AAT AGA TCT CAT TCG CCA TTA CAT ACC ATT TTA ATT GAC (BglII). Each amplified genomic fragment was sequentially subcloned into pUAST vector, followed by the subcloning of *luciferase* to generate a 4X*CG34394*–*luciferase* reporter. For Upd-induced reporter activity, the reporter gene was transfected into S2 cells together with dsRNA for *LacZ* or *Stat92e*. Cells were split into two dishes 3 days after transfection. Half of the cells were co-cultured with S2 cells transfected with the expression plasmid of Upd (Act-Upd) ∼12 hours prior to harvest, and the other half remained untreated as a control [31]. The reporter activity was represented as relative luciferase units (RLU), and was calculated as the ratio of firefly luciferase to *Renilla* luciferase.

### Protein extraction and western blot

Total protein was extracted using RIPA Lysis and Extraction Buffer (Thermo Fisher Scientific, USA) supplemented with Halt^TM^ Protease Inhibitor Cocktail (Life Technologies, USA) and EDTA (Life Technologies, USA). The following antibodies were used: GRAMD1B (Abcam, ab154934), pSTAT3 (Tyr705) (Cell Signaling Technology, #9145), Total STAT3 (Cell Signaling Technology, #12640), Bcl-xL (Cell Signaling Technology, #2762), PARP (Cell Signaling Technology, #9542), β-actin (Sigma- Aldrich, A2228).

### Immunofluorescence staining

Cells were cultured on coverslips and transfected with si-Neg and si-Gramd1b for 72 hours. Cells were fixed in 4% paraformaldehyde, followed by permeabilization using 100% methanol. Cells were then incubated with anti-GRAMD1B antibody (1:100) at 4°C overnight. Alexa Fluor 488 goat anti-rabbit IgG (Thermo Fisher Scientific, USA) was used to detect the primary antibody, and the nucleus was counterstained using 4, 6-diamidino-2-phenylindole (DAPI). The slides were viewed under the Olympus Fluoview FV 1000 Laser Scanning Confocal Microscope.

### Cell proliferation assay

Cell proliferation assay was performed using CellTiter 96^®^ AQ_ueous_ One Solution Cell Proliferation assay (Promega). Cells were transfected with si-Neg or si-Gramd1b for 72 hours, and MTS assay was performed according to manufacturer’s protocol. Absorbance readings were taken after 4 hours of incubation using SpectraMax M5 at an absorbance wavelength of 490nm. Nine readings per well were taken to reduce random error and the average was calculated.

### Cell cycle analysis

Cells were transfected with si-Neg or si-Gramd1b for 48 hours, followed by treatment with AG490 for 24 hours. Cells were harvested, and cell pellets were washed in 1X PBS and fixed in 70% ethanol at 4°C overnight. Cell pellets were then washed in 1X PBS and stained with propidium iodide (PI) cocktail containing 50 μg/ml PI (Sigma-Aldrich) and 0.2 mg/ml RNase A (Roche Applied Science). Cells were subsequently subjected to flow cytometry using BD LSR Fortessa Flow Cytometry Analyser, and the percentages of cells in sub-G1 phase were compared using Summit 3.3 software.

### Acridine orange/ Ethidium bromide (AO/EB) staining

After 48 hours of transfection with si-Neg or si-Gramd1b, cells were treated with AG490 for another 24 hours. The AO/EB dyes were diluted 100-fold in 1X PBS and applied to the cells for 3 minutes in the dark. The cells were then washed with 1X PBS and visualized under the blue excitation filter using the Olympus CKX53 inverted microscope.

### RNA extraction and quantitative real- time polymerase chain reaction

Total RNA was extracted using the RNeasy mini kit (Qiagen GmbH, Germany), followed by cDNA conversion using the Revert Aid First Strand cDNA Synthesis Kit (Thermo Fisher Scientific, USA). FAST SYBR green cocktail from Applied Biosystems (ABI, USA) and primers purchased from IDT technologies were used to conduct PCR analysis using the HT7900 FAST Realtime PCR system from Applied Biosystems. The primers of the genes used for the study are shown in Supplementary Table 2.

### Immunohistochemical staining

Gastric cancer TMA slides were stained for GRAMD1B manually. Following deparaffinization and rehydration of the slides, heat mediated antigen retrieval was carried out using citrate buffer pH 6.0 for 20 minutes, followed by quenching of endogenous peroxidase activity using 3% H_2_O_2_. Anti-GRAMD1B antibody (1:25) was applied overnight at 4°C. Biotinylated anti-rabbit secondary antibody was then applied on the slides for 1 hour at room temperature, followed by Diaminobenzidine (DAB) development and haematoxylin counter-staining for visualization of the nucleus. pSTAT3 (1:25) staining for TMAs was conducted using the Bond Max Automated Immunohistochemistry Vision Biosystem (Leica Microsystems, Germany). The cytoplasmic and nuclear staining was scored separately and verified by a pathologist from Singapore General Hospital. The positive staining was graded into 4 groups: 0 (negative), 1 (weak), 2 (moderate) and 3 (strong) based on intensity of staining, and the scoring was represented as immunoreactive score (IRS), which takes into account both the percentage of stained cells as well as the intensity of staining. Cut off values for positive staining were determined by calculating mean for each group and statistical analysis using PASW Statistics 18 software was carried out.

### Statistical analysis

Statistical analysis was performed using the GraphPad prism 6 software (GraphPad Prism, San Diego, CA, USA). A two-tailed student T-test was used to compare the means between two groups, and one-way ANOVA for more than two groups. A P-value below 0.05 was considered statistically significant, with ^*^, *P* <0.05; ^**^, *P* < 0.01; ^***^, *P* < 0.001; ^****^, *P*< 0.0001 representing significance levels. Data is presented as means with error bars representing SEM of the replicates.

## CONCLUSIONS

We have showed that GRAMD1B is a novel STAT downstream target that may promote gastric tumorigenesis, together with the JAK/STAT cascade. This new knowledge about JAK/STAT-GRAMD1B interaction will provide insights into our understanding of JAK/STAT signaling in gastric cancer.

## SUPPLEMENTARY MATERIALS FIGURE AND TABLES




