# Supplementary material for: GRAM domain-containing protein 1B (GRAMD1B), a novel component of the JAK/STAT signaling pathway, functions in gastric carcinogenesis
Source: Oncotarget. 2017 Dec 15;8(70):115370–83. doi: 10.18632/oncotarget.23265 (PMC5777778; doi:10.18632/oncotarget.23265)
Supplement: Supplementary file 1 [file oncotarget-08-115370-s001.pdf]

GRAM domain-containing protein 1B (GRAMD1B), a novel component of the JAK/STAT signaling pathway, functions in gastric carcinogenesis

SUPPLEMENTARY MATERIALS

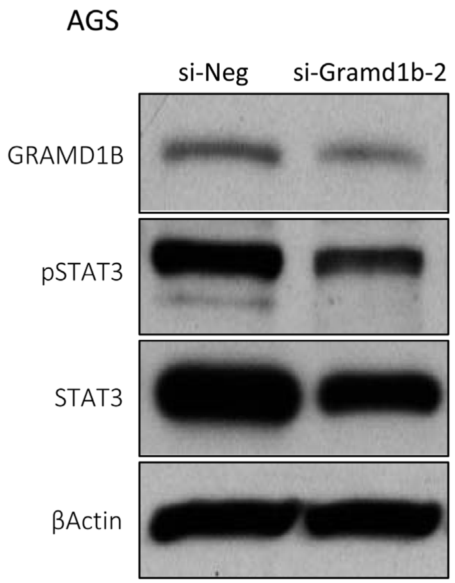

Supplementary Figure 1: si-Gramd1b-2 confirms decrease in total and pSTAT3 levels on GRAMD1B inhibition.

Supplementary Table 1: Clinicopathological parameters of the gastric cancer patient cohort.

See Supplementary File1

Supplementary Table 2: Primers used for quantitative real- time polymerase chain reaction

Gene name	Primers	
	Forward primer	Reverse primer
CG34394	GAG ACG TGC ATT CCA CCT G	CCA ACT GCC TCC TCC AGA
Socs36e	ACG CAA CAC AGC AGC AAG	GGA CAC GGA TGT GGA TGC
RpL11	GGG ATA CCT GTG AGC AGC TT	ATC GCG CTT AAT CTT CTT GG
