# Supplementary material for: GRAM domain-containing protein 1B (GRAMD1B), a novel component of the JAK/STAT signaling pathway, functions in gastric carcinogenesis
Source: Oncotarget. 2017 Dec 15;8(70):115370–83. doi: 10.18632/oncotarget.23265 (PMC5777778; doi:10.18632/oncotarget.23265)
Supplement: Supplementary file 2 [file oncotarget-08-115370-s002.docx]

Supplementary Table 1: Clinicopathological parameters of the gastric cancer patient cohort.

| **Clinicopathological parameters** | **n** | **%** |
| --- | --- | --- |
| **Gender** |  |  |
| **Male** | 42 | 66.7 |
| **Female** | 21 | 33.3 |
| **Age (Years)** |  |  |
| **<=65** | 31 | 49.2 |
| **66+** | 32 | 50.8 |
| **Grade** |  |  |
| **G1** | 5 | 7.9 |
| **G2** | 18 | 28.6 |
| **G3** | 39 | 61.9 |
| **G4** | 1 | 1.6 |
| **Lymph Node status** |  |  |
| **pN0** | 21 | 33.3 |
| **pN1** | 13 | 20.6 |
| **pN2** | 16 | 25.4 |
| **pN3** | 13 | 20.6 |
| **Extent** |  |  |
| **pT1** | 11 | 17.5 |
| **pT2** | 27 | 42.9 |
| **pT3** | 21 | 33.3 |
| **pT4** | 4 | 6.3 |
| **WHO classification** |  |  |
| **Signet Ring** | 15 | 23.8 |
| **Adenocarcinoma** | 18 | 28.6 |
| **Tubular** | 20 | 31.7 |
| **Mucinous** | 1 | 1.6 |
| **Mixed** | 9 | 14.3 |
| **Lauren classification** |  |  |
| **Diffuse** | 21 | 33.3 |
| **Intestinal** | 31 | 49.2 |
| **Mixed** | 11 | 17.5 |
| **Ming classification** |  |  |
| **Infiltrative** | 56 | 88.9 |
| **Expansive** | 7 | 11.1 |
| **Stromal reaction** |  |  |
| **No** | 18 | 28.6 |
| **Yes** | 45 | 71.4 |
| **LVI** |  |  |
| **Absent** | 27 | 42.9 |
| **Present** | 36 | 57.1 |
| **PNI** |  |  |
| **Absent** | 32 | 50.8 |
| **Present** | 31 | 49.2 |
| **Perforation** |  |  |
| **No** | 61 | 96.8 |
| **Yes** | 2 | 3.2 |
